# Trajectories of pain over 6 years in early Parkinson’s disease: ICICLE-PD

**DOI:** 10.1007/s00415-021-10586-7

**Published:** 2021-05-15

**Authors:** J. Naisby, R. A. Lawson, B. Galna, L. Alcock, D. J. Burn, L. Rochester, A. J. Yarnall

**Affiliations:** 1 Department of Sport, Exercise and Rehabilitation, Northumbria University, Newcastle Upon Tyne, UK; 2 Translational and Clinical Research Institute, Newcastle University, Newcastle Upon Tyne, UK; 3 School of Biomedical, Nutritional and Sport Sciences, Newcastle University, Newcastle Upon Tyne, UK; 4 The Newcastle Upon Tyne NHS Foundation Trust, Newcastle Upon Tyne, UK

**Keywords:** Parkinson’s disease, Pain, Longitudinal

## Abstract

**Introduction:**

Pain is a common non-motor symptom in Parkinson’s disease (PD), affecting up to 85% of patients. The frequency and stability of pain over time has not been extensively studied. There is a paucity of high-quality studies investigating pain management in PD. To develop interventions, an understanding of how pain changes over the disease course is required.

**Methods:**

One hundred and fifty-four participants with early PD and 99 age-and-sex-matched controls were recruited as part of a longitudinal study (Incidence of Cognitive Impairment in Cohorts with Longitudinal Evaluation in PD, ICICLE-PD). Pain data were collected at 18-month intervals over 72 months in both groups using the Nonmotor Symptom Questionnaire (NMSQ), consisting of a binary yes/no response. Two questions from the Parkinson’s Disease Questionnaire (PDQ-39) were analysed for the PD group only.

**Results:**

Unexplained pain was common in the PD group and occurred more frequently than in age-matched controls. ‘Aches and pains’ occurred more frequently than ‘cramps and muscle spasms’ at each time point (*p* < 0.001) except 54 months.

**Conclusions:**

This study shows that pain is prevalent even in the early stages of PD, yet the frequency and type of pain fluctuates as symptoms progress. People with PD should be asked about their pain at clinical consultations and given support with describing pain given the different ways this can present.

**Supplementary Information:**

The online version contains supplementary material available at 10.1007/s00415-021-10586-7.

## Introduction

Pain is a common, under recognised symptom in people with Parkinson’s disease (PD) [1] with up to 85% of people with PD reporting pain [2, 3]. Pain influences quality of life and is ranked by people with PD as one of the most troublesome early non-motor symptoms [7]. There have been a number of cross-sectional studies evidencing a significantly higher prevalence of pain in people with PD compared to age-matched controls [4–6]. Currently, the largest study of pain (*n* = 1957) [2], a cross-sectional study, focused on a sample of early stage PD, but to date there have been few longitudinal studies of early PD or with matched controls to explore changes of pain over time. To date, management options are limited, and there is a need for high-quality studies to enhance our understanding to direct therapies to manage pain in PD [8].

To date, no longitudinal studies have focused primarily on pain in PD; however, some measured pain as a small component alongside other non-motor symptoms [10–13] or quality of life [14, 15]. Three studies followed individuals over 2 years and found the presence of pain to be one of the most common symptoms at baseline [10], which could remain relatively stable [13] to significantly worsen with those in the early stages of PD (< 2 years), irrespective of dopaminergic therapy [11]. Within these studies only one measure of pain was collected. Three studies performed a 4-year follow-up and found the presence [15] and frequency of pain to significantly increase [12] and that the increase of pain has a direct impact on cost on disease expenditure over time [14]. However, the stability of pain over time in people with PD was not explored, and pain formed a very limited focus of each of the studies. One study [10] had a control group, but did not measure unexplained pain. Unexplained pain has been highlighted as a problem in PD [16] and warrants further investigation over time to understand how pain evolves. We hypothesised that pain in PD would be more prevalent than age-matched controls over time and that the frequency of pain in PD would increase.

## Methods

The study was approved by the Newcastle and North Tyneside 1 Research Ethics Committee and performed according to the Declaration of Helsinki. All participants provided written informed consent. Recently, diagnosed people with PD were recruited from the community and outpatient clinics in Newcastle upon Tyne and Gateshead from June 1, 2009 to December 31, 2011 as part of the Incidence of Cognitive Impairment in Cohorts with Longitudinal Evaluation in PD (ICICLE-PD) study [17]. Full inclusion criteria have been detailed elsewhere [17]; in brief, newly diagnosed people with PD diagnosed by a movement disorders expert according to the UK Brain Bank criteria were included. Participants returned at 18-month intervals for follow-up evaluation. Age- and sex-matched healthy control subjects were recruited from the local community and underwent a similar assessment schedule as PD participants.

### Assessments

At each assessment, demographic and clinical data were collected including disease severity measured by the Hoehn & Yahr (H&Y) scale, Movement Disorders Society Unified Parkinson’s Disease Rating Scale (MDS-UPDRS) [18] Part III, Geriatric Depression Scale (GDS-15) [19] and levodopa equivalent daily dose (LEDD). Global cognitive function was assessed using the Mini-Mental State Examination (MMSE) [20] and Montreal Cognitive Assessment (MoCA) [21].

Self-reported pain was recorded at 18-month intervals over 72 months using the Nonmotor Symptom Questionnaire (NMSQ) [22], consisting of a binary yes/no response for both the PD and control group. In PD participants, the Parkinson’s Disease Questionnaire (PDQ-39) [23] bodily discomfort domain was analysed as well as two individual items from this domain; ‘had painful muscle cramps or spasms?’ and ‘had aches and pains in your joints or body?’

### Statistical analysis

Statistical analysis was performed using IBM SPSS software (version 22). Demographic characteristics were compared between groups using independent *t* tests, Mann–Whitney *U* tests, and Chi-square statistics, as appropriate. Comparison of the NMSQ for the PD and control group at the five time points was assessed using Chi-squared tests. Repeated-measures testing of categorical variables over time was assessed with Cochran’s *Q*, with post hoc McNemar test, with a Bonferroni adjustment applied to correct for multiple testing. The means from clinical assessments at each time point for the PD group were compared using analyses of variance or Kruskal–Wallis tests, with post hoc Dunn’s test as appropriate. We split these clinical assessments into the presence of unexplained pain from the NMSQ and frequency of painful cramps and muscle spasms and aches and pains from the PDQ-39. The PDQ-39 bodily discomfort domain and the two selected items were compared separately over each time point using Friedman test with a post hoc Wilcoxon signed-rank test and a Bonferroni adjustment applied to correct for multiple testing. The frequency of painful cramps and muscle spasms and aches and pains were compared at each time point using the Wilcoxon signed-rank test and a Bonferroni adjustment applied to correct for multiple testing. Stability of pain over time for unexplained pain, painful cramps, and muscle spasms and aches and pains were calculated descriptively using frequencies.

## Results

**Fig. 1:**
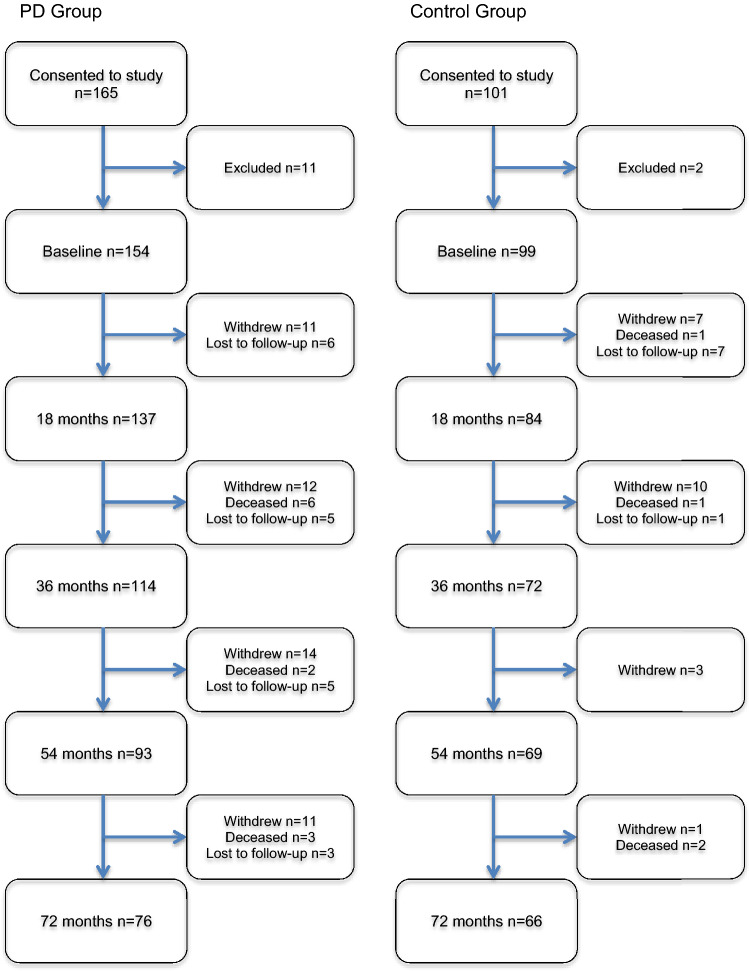
Flow diagram of participants

One hundred and fifty-four newly diagnosed participants with PD and 99 age-matched controls completed baseline assessments and the NMSQ. Figure 1 presents a flowchart of the number of individuals at each time point. Baseline comparisons of both groups are presented in Table 1. PD participants scored significantly lower for global cognition and significantly higher for depression. No other significant differences between the groups were found (*p* > 0.05 for all).

**Table 1:** Baseline characteristics of the PD group and age-matched control group

	PD (*n* = 154)	Controls (*n* = 99)	*p* value
Gender, male, *n* (%)	99 (64.3)	54 (54.5)	0.122^a^
Age, years mean (SD)	67 (10.4)	68 (8.1)	0.270^b^
MDS-UPDRS part lll sub score, mean (SD)	27 (12.1)	n/a	n/a
Hoehn & Yahr stage, *n* (%)			
1	35 (22.7)	n/a	n/a
2	88 (57.1)	n/a	n/a
3	30 (19.5)	n/a	n/a
4	1 (0.6)	n/a	n/a
5	0	n/a	n/a
LEDD (mg/day)	178.0 (148.2)	n/a	n/a
MMSE, mean (SD)	28.6 (1.3)	29 (1.2)	0.006^c^
MoCA^d^ mean (SD)	25 (3.6)	27 (2.5)	**0.001**^c^
GDS-15, mean (SD)	2.8 (2.5)	1.0 (1.5)	**0.001**^c^
Pain diagnoses *n* (%)			
Low back pain	5 (3)	4 (4)	0.739^a^
Osteoarthritis	22 (14)	17 (17)	0.535^a^
Inflammatory arthritis	4 (2)	1 (1)	0.376^a^
Other MSK condition	16 (10)	16 (16)	0.178^a^
Osteoporosis	8 (5)	3 (3)	0.410^a^
Joint replacement	10 (6)	13 (13)	0.073^a^
Adult fracture	8 (5)	5 (5)	0.960^a^

Bold indicates a significant difference

^a^*χ*^2^

^b^Independent *t* test

^c^Mann–Whitney *U* test

^d^97/99 control group completed and 140/154 PD group completed

### Unexplained pain (NMSQ)

The PD group reported more unexplained pain than the control group, with this being statistically significant at each time point except 36 months (baseline: 3% vs. 38%, respectively, *p* < 0.001; 18 mo: 2% vs. 45%, respectively, *p* < 0.0001 36 mo 17% vs. 29%, respectively *p* = 0.057; 54 mo 16% vs. 36%, respectively, *p* = 0.005; 72 mo 5% vs. 33%, respectively, *p* < 0.001).

#### Stability of pain over time in PD: unexplained pain

**Fig. 2:**
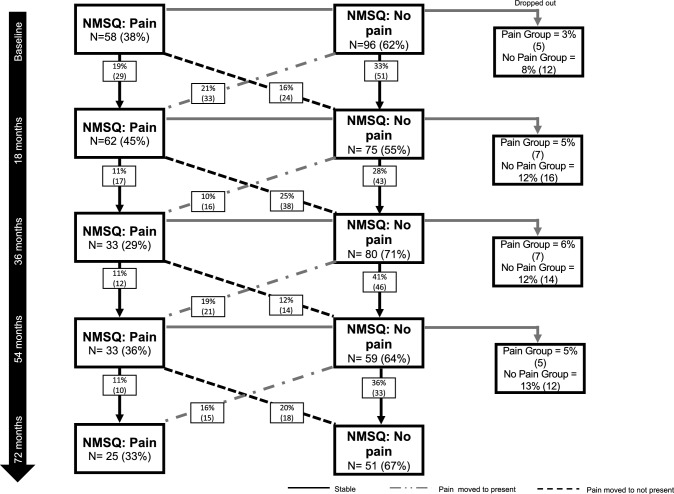
Changes in unexplained pain from baseline to 72 months

Cochran’s Q test did not indicate any significant difference across the five time points (62% vs. 55% vs. 71% vs. 64% vs. 67%, respectively, *p* = 0.76) for the NMSQ for the PD group. 18–36 months demonstrated the largest percentage change with participants moving from pain to no pain (61% of the pain group). Unexplained pain fluctuated among the group. Of the overall sample, 2% had pain consistently at each time point, 9% consistently had no pain over time, and the remaining 38% fluctuated between yes and no up to 72 months. Between 29 and 45% of the sample experienced unexplained pain at each time point. Figure 2 presents the stability of unexplained pain over time from NMSQ in the PD group.

Table 2 reports the clinical characteristics of those with and without unexplained pain. At 18 months (*p* = 0.006) and 36 months (*p* < 0.001), participants were significantly younger who reported pain. Females were significantly more likely to experience unexplained pain than males at baseline (*p* = 0.029), 36 (*p* = 0.040), and 54 months (*p* = 0.001). MDS-UPDRS III scores, Hoehn and Yahr stage or LEDD were not significantly different at any time point (*p* > 0.05 for all) between the presence and absence of unexplained pain.

**Table 2:** Clinical characteristics of PD group and the NMSQ presence of unexplained pain

	Baseline (*n* = 154)			18 months (*n* = 137)			36 months (*n* = 113)			54 months (*n* = 92)			72 months (*n* = 76)		
	Unexplained pain (*n* = 58)	No unexplained pain (*n* = 96)	*p* value	Unexplained pain (*n* = 62)	No unexplained pain (*n* = 65)	*p* value	Unexplained pain (*n* = 33)	No unexplained pain (*n* = 80)	*p* value	Unexplained pain (*n* = 33)	No unexplained pain (*n* = 59)	*p* value	Unexplained pain (*n* = 25)	No unexplained pain (*n* = 51)	*p* value
Age (years)	65 (11.9)	68 (9.3)	0.145	66 (10)	71 (9)	**0.006**	64 (10.8)	72 (8.5)	** < 0.001**	70 (9)	70 (10.1)	0.829	71 (10.1)	71 (9.7)	0.786
Gender: female *n* (%)	27 (46.6)	28 (29.2)	**0.029**^**a**^	27 (44.5)	21 (32.3)	0.058^a^	16 (48.5)	23 (28.7)	**0.040**^a^	17 (51.5)	11 (18.6)	**0.001**^a^	8 (32)	16 (31.4)	0.956^a^
MDS-UPDRS lll	27.0 (11.7)	26.9 (12.4)	0.909	32.8 (11.3)	35.0 (12.0)	0.377	38.0 (15.4)	38.8 (12.3)	0.713	38.5 (13.9)	40.9 (13.6)^†^	0.392	41.4 (13.9)	41.3 (14.1)	0.903
Hohen and Yahr	2.1 (0.7)	1.9 (0.7)	0.240	2.2 (0.5)	2.2 (0.6)	0.735	2.1 (0.5)	2.2 (0.5)	0.217	2.2 (0.7)	2.2 (0.6)	0.689	2.4 (0.9)	2.4 (0.6)	0.491
LEDD (mg/day)	179.0 (129.7)	177.5 (158.9)	0.535	400.3 (202.9)	392.3 (217.8)	0.749	498.6 (275.2)	523.6 (254.1)	0.467	575.6 (225.2)	705.4 (318.6)	0.085	725.2 (348.7)	731.5 (320.6)	0.864
GDS-15	3.1 (3.0)	2.7 (2.2)	0.729	2.9 (3.0)	2.9 (2.8)	0.773	2.9 (2.8)	2.9 (2.4)	0.829	2.5 (2.0)	3.1 (3.3)	0.880	3.4 (3.3)	3.1 (2.8)	0.823
MOCA^*^	25.1 (3.7)	25.2 (3.6)	0.766	26.0 (4.0)	25.6 (3.6)	0.205	26.0 (4.7)	25.5 (3.6)	0.205	25.8 (4.3)	25.0 (4.5)	0.330	24.4 (5.4)	24.6 (4.5)	0.894
MMSE	28.5 (1.3)	28.7 (1.3)	0.202	28.5 (1.6)	28.2 (1.9)	0.459	27.8 (3.1)	28.2 (2.0)	0.950	27.8 (2.5)	27.1 (3.2)^‡^	0.234	26.7 (3.8)	27.4 (2.9)	0.489

Figures are mean (SD) unless otherwise stated, significant differences are highlighted in bold. Statistical tests are Mann–Whitney *U* Test unless otherwise stated

^*^n = 24 did not complete MoCA at baseline, *n* = 3 did not complete at 18 months, *n* = 4 at 36 months, and *n* = 1 at 54 months

^†^*n* = 1 did not complete MDS-UPDRS at 54 months

^‡^*n* = 2 did not complete MMSE at 54 months

^a^*χ*^2^

### PDQ-39 bodily discomfort

One hundred and fifty-three of the 154 newly diagnosed individuals with PD completed the PDQ-39 at baseline. Table 3 presents the number completed at each time point. The frequency of bodily discomfort increased over time (*p* < 0.001), and the frequency of participants reporting ‘muscle cramps or spasms’ (Q37) increased over time (*p* ≤ 0.005); however, ‘aches and pains’ in your joints or body? (Q38) demonstrated no significant change over time (Bonferroni correction *p* > 0.005). The item scores of painful muscle spasms and aches and pains were compared at each time point, demonstrating that aches and pains were more frequent pain at all time points (*p* < 0.01 for all) except 54 months (*p* > 0.05). No participant consistently reported ‘never’ for experiencing aches and pains (Supplementary materials). Instead, these symptoms fluctuated, with the largest percentage increase in frequency between baseline and 18 months (9%). The frequency of painful muscle cramps and spasms increased over time (Supplementary materials) with only 2% of individuals having never experienced muscle spasms and cramps over time.

**Table 3:** Clinical characteristics of PD participants by pain frequency responses over 72 months

	Baseline *n* = 153				18 months *n* = 135				36 months *n* = 107				54 months *n* = 89				72 months *n* = 76			
	never	Occasionally-sometimes	Often-always	*p* value	Never	Occasionally-sometimes	Often-always	*p* value	Never	Occasionally-sometimes	Often-always	*p* value	Never	Occasionally-sometimes	Often-always	*p* value	Never	Occasionally-sometimes	Often-always	*p* value
*n*																				
Cramps/spasms	69	58	26		38	65	32		27	60	20		22	45	22		19	36	21	
Aches/pains	38	72	43		25	61	49		15	58	34		15	43	31		9	40	27	
Age (years)^a^																				
Cramps/spasms	68.7 (8.2)	65.7 (11.3)	62.8 (12.1)	**0.031**	69.9 (9.2)	69.2 (9.3)	66.3 (11.8)	0.271	73.9 (7.9)	68.4 (9.0)	65.1 (10.1)	**0.005**	69.6 (9.1)	72.2 (8.6)	64.3 (10.1)	**0.005**	73.8 (9.2)	72.8 (8.2)	65.2 (10.7)	**0.005**
Aches/pains	68.2 (7.1)	67.3 (10.6)	64.0 (12.0)	0.151	70.1 (8.1)	69.4 (8.8)	67.2 (11.8)	0.358	71.2 (7.3)	69.2 (9.1)	68.2 (12.1)	0.618	69.2 (7.3)	71.4 (9.7)	67.4 (10.1)	0.196	73.8 (8.2)	73.7 (8.8)	65.9 (9.8)	0.003
Gender: female *n* (%)^b^																				
Cramps/spasms	19 (27.5)	16 (27.6)	19 (73.1)	** < 0.001**	11 (28.9)	24 (36.9)	13 (40.6)	0.567	9 (33.3)	18 (30.0)	9 (45.0)	0.469	8 (36.4)	13 (28.9)	7 (31.8)	0.825	8 (42.1)	9 (25.0)	7 (33.3)	0.422
Aches/pains	11 (29.9)	23 (32.0)	20 (46.6)	0.183	6 (24.0)	21 (34.4)	21 (42.9)	0.268	4 (26.7)	21 (36.2)	11 (32.4)	0.770	8 (53.3)	11 (25.6)	9 (29.0)	0.129	3 (33.3)	12 (30.0)	9 (33.3)	0.952
MDS-UPDRS lll																				
Cramps/spasms	23.5 (10.7)	30.2 (11.9)	27.8 (12.0)	**0.012**	30.7 (10.5)	33.8 (11.8)	37.1 (10.9)	0.051	36.4 (12.0)	37.9 (13.2)	42.1 (14.4)	0.439	34 (7.8)	43.6 (13.4)	38.9 (17.3)	**0.030**	40.3 (15.1)	40.8 (11.6)	43.1 (16.8)	0.678
Aches/pains	24.1 (11.7)	25.9 (11.8)	30.6 (11.9)	**0.028**	30.2 (12.3)	31.3 (9.5)	38.5 (11.7)	**0.002**	31.9 (13.8)	37.2 (12.1)	43.1 (13.3)	**0.019**	33.9 (10.2)	40.3 (11.9)	42.7 (16.9)	0.153	46.4 (16.2)	41.0 (13.0)	40.2 (14.6)	0.621
LEDD (mg/day)																				
Cramps/spasms	174.4 (129.8)	191.6 (178.5)	152.8 (119.1)	0.769	364.5 (180.2)	399.4 (207.4)	421.3 (239.9)	0.658	465.4 (221.9)	512.4 (258.9)	567.7 (297)	0.369	583.3 (157.8)	626.4 (305.5)	782.8 (349.2)	0.140	690.6 (252.9)	673.0 (293.3)	861.4 (411. 5)	0.323
Aches/pains	146.7 (130.2)	170.7 (145.7)	215.1 (162.5)	0.084	394.0 (146.3)	377.5 (206.9)	416.6 (236.1)	0.513	491.6 (154.7)	485.1 (245.5)	563.2 (308.2)	0.515	611.4 (207.8)	602.0 (261.7)	747.9 (355.9	0.225	583.9 (179.7)	645.4 (198.7)	902.5 (437. 6)	0.03
GDS-15																				
Cramps/spasms	2.3 (2.0)	3.1 (2.6)	3.7 (3.2)	0.090	1.8 (1.9)	2.9 (3.1)	4.3 (3.2)	**0.002**	2.6 (2.2)	2.7 (2.5)	3.9 (3)	0.188	1.8 (2.1)	2.9 (2.9)	3.9 (3.2)	**0.012**	2.5 (2.8)	2.6 (2.6)	4.8 (3.3)	**0.007**
Aches/pains	2.1 (1.7)	2.6 (2.5)	3.8 (2.9)	**0.011**	2.2 (2.7)	2.0 (2.3)	4.4 (3.2)	** < 0.001**	2.7 (2.1)	2.4 (2.4)	3.8 (2.8)	**0.035**	1.7 (2.4)	2.2 (2.1)	4.4 (3.4)	**0.001**	3.1 (3.2)	2.9 (2.6)	3.8 (3.3)	0.410
MOCA^*^																				
Cramps/spasms	25.5 (3.1)	24.6 (4.1)	25.6 (4.0)	0.443	26.9 (3.1)	25.4 (4.0)^†^	25.4 (3.8)	0.095	26.0 (3.5)	25.6 (4.1)	26.0 (4.2)	0.797	27.5 (2.6)^‡^	24.4 (5.0)	24.9 (4.1)	**0.016**	25.4 (5.5)	24.8 (4.3)	24.1 (5.1)	0.949
Aches/pains	25.1 (3.8)	25.4 (3.2)	24.9 (4.2)	0.994	26.1 (4.1)	26.3 (3.6)^†^	25.2 (3.9)	0.268	26.1 (3.8)	26.1 (3.5)	25.1 (3.9)	0.732	27.2 (2.3)	24.9 (4.7)	24.8 (4.7)	0.225	25.0 (5.5)	24.3 (4.5)	24.7 (4.8)	0.554

Figures are mean (SD) unless otherwise stated, significant differences are highlighted in bold after correction for multiple comparison. Between group differences were assessed using Kruskal–Wallis test unless otherwise specified

^*^n = 14 did not complete MoCA at baseline

^†^*n* = 60 participants

^‡^*n* = 13 participants

^a^ANOVA

^b^*χ*^2^

Table 3 presents the demographic and clinical characteristics of PD participants at each time point for the individual pain items (Q37 and Q38) of the PDQ-39. Those who had a higher frequency of muscle cramps and spasms at each time point were younger, except 18 months. There were no significant differences between the frequency of muscle cramps and spasms and MDS-UPDRS lll at any time point except baseline. There was a significant difference between never experiencing aches and pains to often to always experiencing these between 0 and 18 and 18–36 months for the MDS-UPDRS lll score. There was no significant difference between the painful muscle cramps and spasms groups according to H&Y stage. The H&Y stage was significantly higher as pain frequency increased between the aches and pains group for baseline (*p* = 0.005). There was no significant difference between LEDD and frequency of either muscle cramps and spasms or aches and pains. Depression (GDS-15) at baseline, 18 and 36 months demonstrated a significant difference, with higher scores for the often-always groups of both.

## Discussion

We have shown that unexplained pain is common across the early stages of PD and occurs more frequently than in age-matched controls. To our knowledge, this is the first study to explore the presence, frequency, and trajectory of pain in PD over 72 months.

Whilst it is known from cross-sectional studies that pain often occurs more frequently in people with PD than age-matched control group [4], few longitudinal studies that capture pain have included a comparator. One longitudinal study exploring non-motor symptoms over 2 years reported that the severity of ‘pain and other sensations,’ measured by a single item on the MDS-UPDRS, was significantly more severe in the PD compared to age-matched controls [10]. The current study enhances our understanding of the presence of unexplained pain specifically being significantly higher in the PD group over time, as well as consideration of a longer time period. This is an important finding, as it has been identified that people with PD are uncertain about raising the problem of unexplained pain to healthcare professionals due to uncertainty regarding the link to PD and uncertainty surrounding treatment despite facing daily challenges with this symptom [24].

Our study identified that between 29 and 45% of people with PD reported unexplained pain at each assessment. This finding is in agreement with a longitudinal study exploring the progression of non-motor symptoms over 2 years, in which unexplained pain was approximately 40% at each time point [13]. Our findings highlight the presence of unexplained pain fluctuates among individuals over a longer time frame and develops our understanding of the trajectory of this type of pain with only 2% of individuals consistently reporting this symptom at each time point. Thus, this symptom may be reported for the first time or change at various stages during the course of PD, highlighting the importance of screening for unexplained pain at routine clinical consultations [24]. A previous cross-sectional study highlighted the role of central processes in pain within early/moderate PD [2] and encouraged moving away from a focus on peripheral processes. Pain has been highlighted as one of the most bothersome non-motor symptoms in early PD [7] and, this study highlights that unexplained pain symptoms are common even in early PD. Of note, the NMSQ captured unexplained pain. Chronic pain is known to recur and should not be seen as discrete events [25]. The time of reporting may also influence the responses, and may be prone to recall bias, with the response only applying to the specific time the measure was taken. A yes/no response does not capture the individual’s history of pain, the severity or frequency of the pain, and their longer term trajectory [25]. Prognostic factors for pain, such as pain intensity and beliefs that pain will last into the future, have been associated with poor outcome in low back pain [26], but such associations have not been explored in PD. Future studies should aim to capture a wider range of factors to understand pain reporting in PD, and prognostic factors.

The presence or absence of unexplained pain was not associated with motor severity (MDS-UPDRS III), disease stage (H&Y), or LEDD, corroborating findings from a shorter longitudinal study [12]. Currently, the link between pain and motor symptoms is unclear [27] and exploration of the different descriptions of pain as captured within our study can develop understanding of reporting of symptoms at different time points.

The PDQ-39 bodily discomfort domain was used in the current study to determine self-reported frequency of pain. The different descriptions of pain through two of these questions allowed detailed exploration of their relationship with other symptoms. An increased frequency of aches and pains was associated with an increased MDS-UPDRS lll score up to 36 months, whereas ‘occasional to sometimes’ muscle spasms or cramps was associated with higher MDS-UPDRS lll scores at baseline and 54 months, but not ‘always to frequent’ symptoms. The reporting of aches and pains remained fairly consistent across the different time points, albeit with fluctuations among individual participants. However, reporting of painful muscle cramps and spasms changed; although 45% never experienced this at baseline, only 2% never experienced this over 6 years. The highest percentage (28%) of individuals experiencing muscle cramps and spasms ‘often to always’ was observed at the final time point; 72 months. How individuals describe their pain is relevant to consider during clinical consultations. Individuals may experience different types of pain at different times, and these may not be mutually exclusive [30]. During pain assessments, the different descriptions and types of pain need to be captured [27] as demonstrated in the present study.

Up to 36 months, there was a trend for individuals experiencing unexplained pain and increased frequency of aches and pains or muscle spasms and cramp to be younger. This finding is in agreement with current cross-sectional studies, highlighting individuals with pain are often of a younger age, and younger age at onset being linked with pain in PD [2, 27, 28]. Geriatric depression score followed a trend to be higher in the more frequent pain groups of the PDQ-39 at each time point, with this finding echoing current cross-sectional studies exploring the association between depression, pain and PD [2, 28].

Longitudinal studies often report only one measure of pain [10, 11, 13]. Erro et al. explored the same measures as in the present study, which focused on non-motor symptoms and quality of life over a 4-year period [12]. The bodily discomfort domain of the PDQ-39 worsened over time, indicating an increased frequency of this symptom. However, this longitudinal study [12] provided very limited detail regarding pain. The present study has provided detail of how pain is described over time by people with PD and developed understanding of the trajectory. A number of cross-sectional studies classify the type of pain experienced by people with PD, with musculoskeletal pain highlighted as the most frequent [28, 29] and documenting the different types of pain reported by people with PD [30]. Further research is required longitudinally using measures such as the Kings Parkinson’s Pain Scale [31], which capture the type of pain people with PD experience in more greater detail using consistent measuring tools and classifications to allow comparisons across the literature.

The key strength of this study is that a large cohort of patients with newly diagnosed PD and age-matched controls were recruited and followed longitudinally over 6 years. Other strengths include the use of validated instruments for the assessment of motor symptoms and NMSQ and the measures of pain being validated in a PD group. This study does have several limitations. A number of participants did not return for further assessments; these participants may have been important to the findings of this study. It is possible that these patients may have reported a higher burden of pain. However, on exploration of drop out from each of the pain groups, the trend remained the same over time for each of the pain questions (data not shown). We did not use a specific measure of pain within this study or explore the use of pain medication, which should be investigated in future studies.

In conclusion, pain in PD is prevalent in the early stages of the disease, and the type and frequency of pain people with PD experience fluctuates. People with PD should be asked about their pain at each clinical consultation and given support with describing this given the different ways this can present.

## Supplementary Information

Below is the link to the electronic supplementary material.

Supplementary file1 (PPTX 59 KB)

Supplementary file2 (PPTX 59 KB)
