# Supplementary material for: Trajectories of pain over 6 years in early Parkinson’s disease: ICICLE-PD
Source: J Neurol. 2021 May 15;268(12):4759–67. doi: 10.1007/s00415-021-10586-7 (PMC8563518; doi:10.1007/s00415-021-10586-7)
Supplement: Supplementary file 1 — Supplementary file1 (PPTX 59 KB) [file 415_2021_10586_MOESM1_ESM.pptx]

## Slide 1
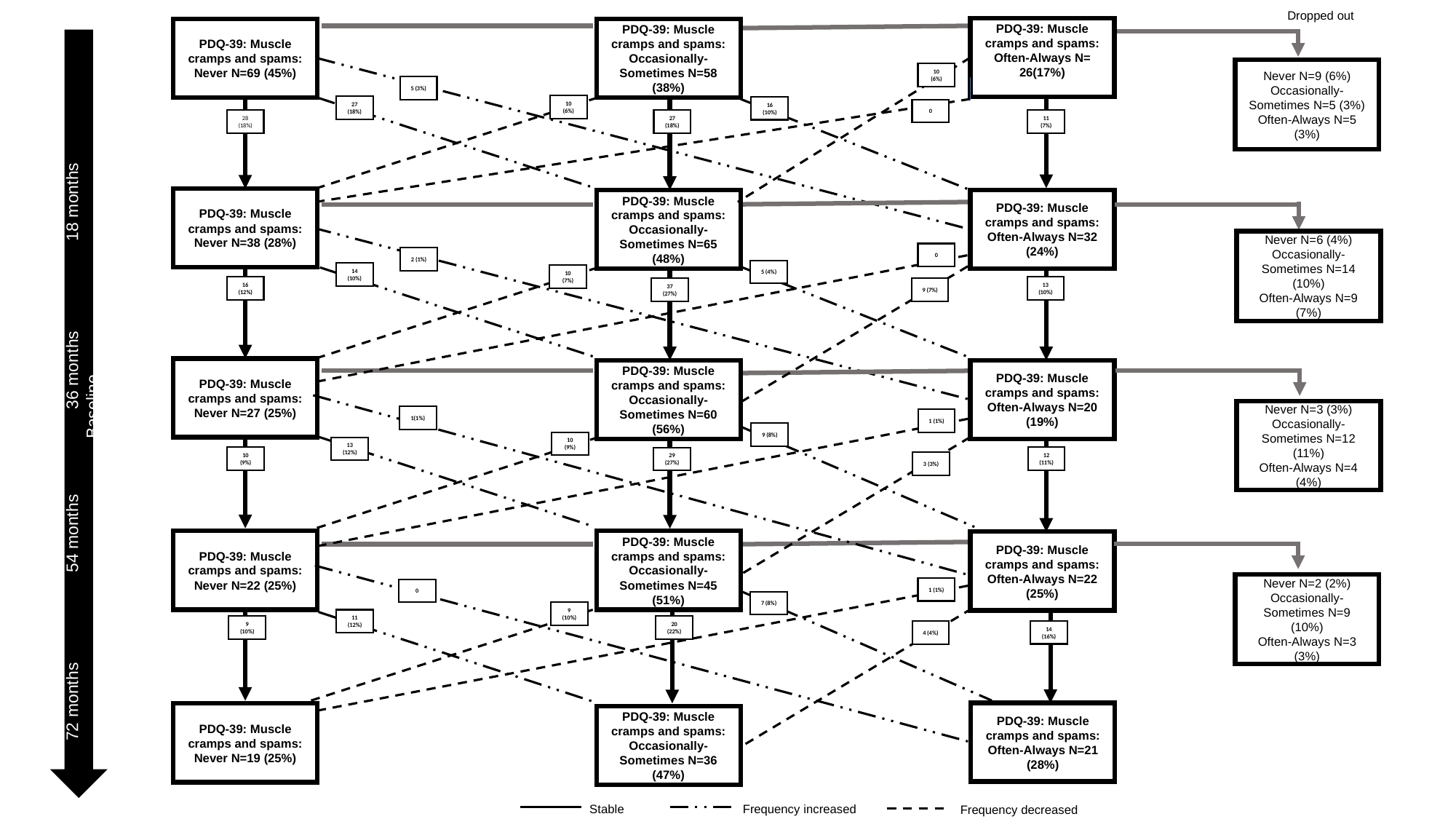

Dropped out
PDQ-39: Muscle cramps and spams: Often-Always N= 26(17%)
PDQ-39: Muscle cramps and spams: Never N=69 (45%)
PDQ-39: Muscle cramps and spams: Occasionally-Sometimes N=58 (38%)
72 months 54 months 36 months 18 months Baseline
Never N=9 (6%)
Occasionally-Sometimes N=5 (3%)
Often-Always N=5 (3%)
10 (6%)
5 (3%)
10 (6%)
27 (18%)
16 (10%)
0
28 (18%)
27 (18%)
11 (7%)
PDQ-39: Muscle cramps and spams: Never N=38 (28%)
PDQ-39: Muscle cramps and spams: Occasionally-Sometimes N=65 (48%)
PDQ-39: Muscle cramps and spams: Often-Always N=32 (24%)
Never N=6 (4%)
Occasionally-Sometimes N=14 (10%)
Often-Always N=9 (7%)
0
2 (1%)
5 (4%)
14 (10%)
10 (7%)
16
(12%)
13 (10%)
37 (27%)
9 (7%)
PDQ-39: Muscle cramps and spams: Never N=27 (25%)
PDQ-39: Muscle cramps and spams: Occasionally-Sometimes N=60 (56%)
PDQ-39: Muscle cramps and spams: Often-Always N=20 (19%)
Never N=3 (3%)
Occasionally-Sometimes N=12 (11%)
Often-Always N=4 (4%)
1(1%)
1 (1%)
9 (8%)
10 (9%)
13 (12%)
10 (9%)
12 (11%)
29 (27%)
3 (3%)
PDQ-39: Muscle cramps and spams: Never N=22 (25%)
PDQ-39: Muscle cramps and spams: Occasionally-Sometimes N=45 (51%)
PDQ-39: Muscle cramps and spams: Often-Always N=22 (25%)
Never N=2 (2%)
Occasionally-Sometimes N=9 (10%)
Often-Always N=3 (3%)
1 (1%)
0
7 (8%)
9 (10%)
11 (12%)
9 (10%)
20 (22%)
14 (16%)
4 (4%)
PDQ-39: Muscle cramps and spams: Often-Always N=21 (28%)
PDQ-39: Muscle cramps and spams: Never N=19 (25%)
PDQ-39: Muscle cramps and spams: Occasionally-Sometimes N=36 (47%)
Stable
Frequency increased
Frequency decreased
