# Supplementary material for: Trajectories of pain over 6 years in early Parkinson’s disease: ICICLE-PD
Source: J Neurol. 2021 May 15;268(12):4759–67. doi: 10.1007/s00415-021-10586-7 (PMC8563518; doi:10.1007/s00415-021-10586-7)
Supplement: Supplementary file 2 — Supplementary file2 (PPTX 59 KB) [file 415_2021_10586_MOESM2_ESM.pptx]

## Slide 1
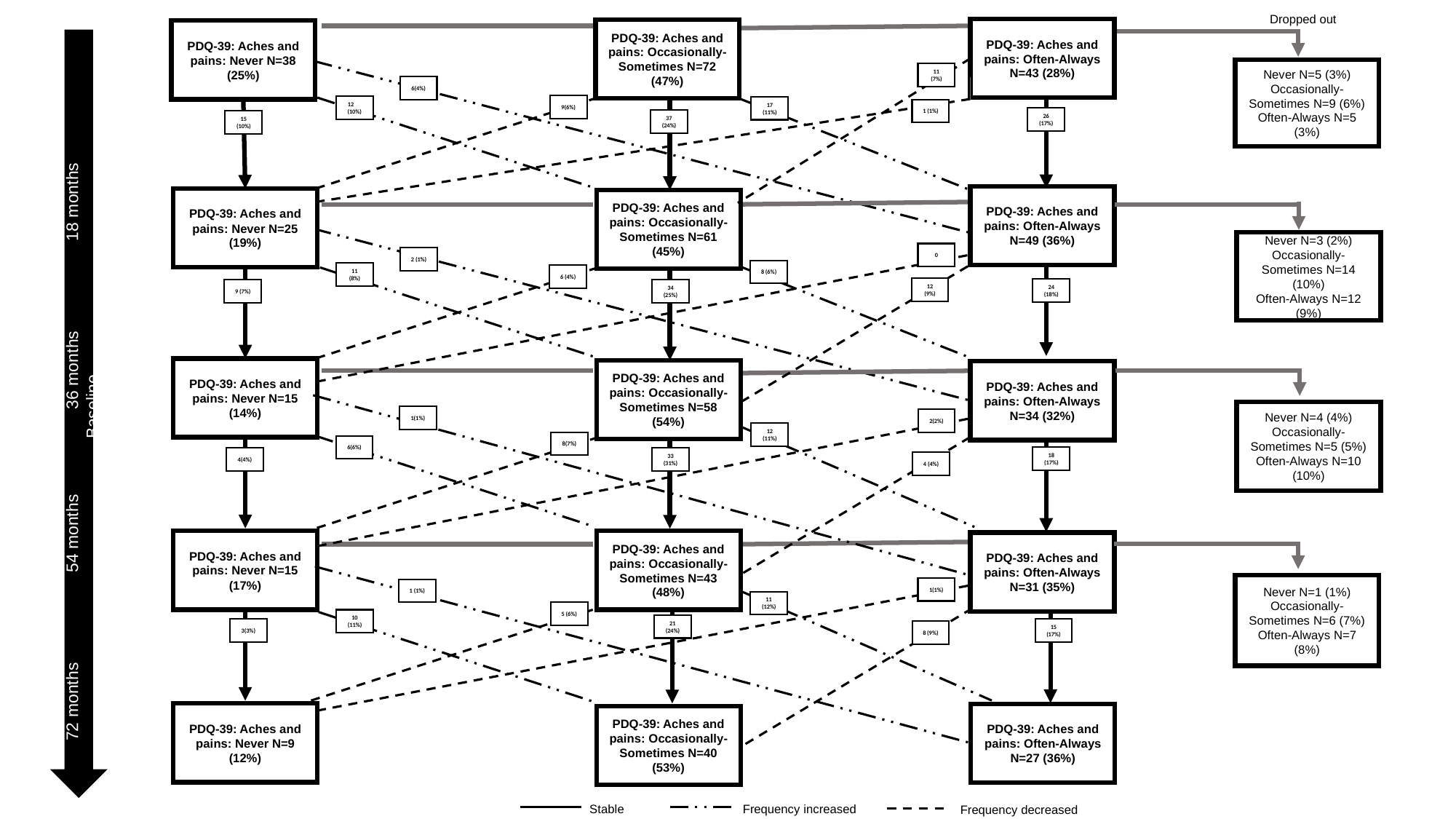

Dropped out
PDQ-39: Aches and pains: Often-Always N=43 (28%)
PDQ-39: Aches and pains: Occasionally-Sometimes N=72 (47%)
PDQ-39: Aches and pains: Never N=38 (25%)
72 months 54 months 36 months 18 months Baseline
Never N=5 (3%)
Occasionally-Sometimes N=9 (6%)
Often-Always N=5 (3%)
11 (7%)
6(4%)
9(6%)
12 (10%)
17 (11%)
1 (1%)
26 (17%)
37 (24%)
15 (10%)
PDQ-39: Aches and pains: Often-Always N=49 (36%)
PDQ-39: Aches and pains: Never N=25 (19%)
PDQ-39: Aches and pains: Occasionally-Sometimes N=61 (45%)
Never N=3 (2%)
Occasionally-Sometimes N=14 (10%)
Often-Always N=12 (9%)
0
2 (1%)
8 (6%)
11 (8%)
6 (4%)
12 (9%)
24 (18%)
9 (7%)
34 (25%)
PDQ-39: Aches and pains: Never N=15 (14%)
PDQ-39: Aches and pains: Occasionally-Sometimes N=58 (54%)
PDQ-39: Aches and pains: Often-Always N=34 (32%)
Never N=4 (4%)
Occasionally-Sometimes N=5 (5%)
Often-Always N=10 (10%)
1(1%)
2(2%)
12 (11%)
8(7%)
6(6%)
18 (17%)
4(4%)
33 (31%)
4 (4%)
PDQ-39: Aches and pains: Never N=15 (17%)
PDQ-39: Aches and pains: Occasionally-Sometimes N=43 (48%)
PDQ-39: Aches and pains: Often-Always N=31 (35%)
Never N=1 (1%)
Occasionally-Sometimes N=6 (7%)
Often-Always N=7 (8%)
1(1%)
1 (1%)
11 (12%)
5 (6%)
10 (11%)
21 (24%)
3(3%)
15 (17%)
8 (9%)
PDQ-39: Aches and pains: Never N=9 (12%)
PDQ-39: Aches and pains: Often-Always N=27 (36%)
PDQ-39: Aches and pains: Occasionally-Sometimes N=40 (53%)
Stable
Frequency increased
Frequency decreased
